# Supplementary material for: Autophagic- and Lysosomal-Related Biomarkers for Parkinson’s Disease: Lights and Shadows
Source: Cells. 2019 Oct 25;8(11):1317. doi: 10.3390/cells8111317 (PMC6912814; doi:10.3390/cells8111317)
Supplement: Supplementary file 1 [file cells-08-01317-s001.pdf]

("autophagy"[MeSH Terms] OR "autophagy"[All Fields] OR "lysosomes"[MeSH Terms] OR "lysosomes"[All Fields] OR (lysosomal[All Fields] AND ("enzymes"[MeSH Terms] OR "enzymes"[All Fields] OR "enzyme"[All Fields])) OR "mitochondrial degradation"[MeSH Terms] OR ("mitochondrial"[All Fields] AND "degradation"[All Fields]) OR "mitochondrial degradation"[All Fields] OR "mitophagy"[All Fields]

OR

(AGA[All Fields] OR ("aspartylglucosylaminase"[MeSH Terms] OR "aspartylglucosylaminase"[All Fields] OR "aspartylglucosaminidase"[All Fields])) OR (ARSA[All Fields] OR ("arylsulphatase a"[All Fields] OR "cerebroside-sulfatase"[MeSH Terms] OR "cerebroside-sulfatase"[All Fields] OR "arylsulfatase a"[All Fields])) OR (ARSB[All Fields] OR ("arylsulphatase b"[All Fields] OR "n-acetylgalactosamine-4-sulfatase"[MeSH Terms] OR "n-acetylgalactosamine-4-sulfatase"[All Fields] OR "arylsulfatase b"[All Fields])) OR (ASAH1[All Fields] OR ("ceramidases"[MeSH Terms] OR "ceramidases"[All Fields] OR "n acylsphingosine amidohydrolase"[All Fields])) OR ATP13A2[All Fields] OR CLN3[All Fields] OR CLN5[All Fields] OR CLN6[All Fields] OR CLN8[All Fields] OR (CTNS[All Fields] OR cystinosin[All Fields]) OR (CTSA[All Fields] OR "cathepsin a"[MeSH Terms] OR "cathepsin a"[All Fields]) OR (CTSD[All Fields] OR "cathepsin d"[MeSH Terms] OR "cathepsin d"[All Fields]) OR (CTSF[All Fields] OR "cathepsin f"[MeSH Terms] OR "cathepsin f"[All Fields]) OR (CTSF[All Fields] OR "cathepsin f"[MeSH Terms] OR "cathepsin f"[All Fields]) OR DNAJC5[All Fields] OR (FUCA1[All Fields] OR (("alpha-l-fucosidase"[MeSH Terms] OR "alpha-l-fucosidase"[All Fields] OR "alpha l fucosidase"[All Fields]) AND 1[All Fields])) OR (GAA[All Fields] OR (("glucosidases"[MeSH Terms] OR "glucosidases"[All Fields] OR "glucosidase"[All Fields]) AND alpha[All Fields] AND ("acids"[MeSH Terms] OR "acids"[All Fields] OR "acid"[All Fields]))) OR (GALC[All Fields] OR "galactosylceramidase"[MeSH Terms] OR "galactosylceramidase"[All Fields]) OR (GALNS[All Fields] OR (("galactosamine"[MeSH Terms] OR "galactosamine"[All Fields]) AND N-acetyl[All Fields] AND ("sulfatases"[MeSH Terms] OR "sulfatases"[All Fields] OR "6 sulfatase"[All Fields]))) OR (GBA[All Fields] OR (("glucosylceramidase"[MeSH Terms] OR "glucosylceramidase"[All Fields]) AND "beta"[All Fields])) OR (GLA[All Fields] OR (("galactosidases"[MeSH Terms] OR "galactosidases"[All Fields] OR "galactosidase"[All Fields]) AND alpha[All Fields])) OR (GLB1[All Fields] OR (("galactosidases"[MeSH Terms] OR "galactosidases"[All Fields] OR "galactosidase"[All Fields]) AND ("beta"[All Fields]) AND 1[All Fields])) OR (GM2A[All Fields] OR (("g(m2) ganglioside"[MeSH Terms] OR ("g(m2)"[All Fields] AND "ganglioside"[All Fields]) OR "g(m2) ganglioside"[All Fields] OR ("gm2"[All Fields] AND "ganglioside"[All Fields]) OR "gm2 ganglioside"[All Fields]) AND activator[All Fields])) OR (GNPTAB[All Fields] OR (("UDPacetylglucosamine-dolichyl-phosphate acetylglucosamine-1-phosphate transferase"[Supplementary Concept] OR "UDPacetylglucosamine-dolichyl-phosphate acetylglucosamine-1-phosphate transferase"[All Fields] OR "n acetylglucosamine 1 phosphate transferase"[All Fields]) AND subunits[All Fields] AND alpha[All Fields] AND "beta"[All Fields])) OR (GNS[All Fields] OR (("glucosamine"[MeSH Terms] OR "glucosamine"[All Fields]) AND N-acetyl[All Fields] AND ("sulfatases"[MeSH Terms] OR "sulfatases"[All Fields] OR "6 sulfatase"[All Fields]))) OR (GRN[All Fields] OR ("progranulins"[MeSH Terms] OR "progranulins"[All Fields] OR ("granulin"[All Fields] AND "precursor"[All Fields]) OR "granulin precursor"[All Fields])) OR (GUSB[All Fields] OR (("glucuronidase"[MeSH Terms] OR "glucuronidase"[All Fields]) AND ("beta"[All Fields]))) OR (HEXA[All Fields] OR (("hexosaminidases"[MeSH Terms] OR "hexosaminidases"[All Fields] OR "hexosaminidase"[All

Fields] OR "beta-n-acetylhexosaminidases"[MeSH Terms] OR "beta-n-acetylhexosaminidases"[All Fields]) AND ("protein subunits"[MeSH Terms] OR ("protein"[All Fields] AND "subunits"[All Fields]) OR "protein subunits"[All Fields] OR "subunit"[All Fields]) AND alpha[All Fields])) OR (HEXB[All Fields] OR (("hexosaminidases"[MeSH Terms] OR "hexosaminidases"[All Fields] OR "hexosaminidase"[All Fields] OR "beta-n-acetylhexosaminidases"[MeSH Terms] OR "beta-n-acetylhexosaminidases"[All Fields]) AND ("protein subunits"[MeSH Terms] OR ("protein"[All Fields] AND "subunits"[All Fields]) OR "protein subunits"[All Fields] OR "subunit"[All Fields]) AND ("beta"[All Fields]))) OR (HGSNAT[All Fields] OR (heparan-alpha-glucosaminide[All Fields] AND N-acetyltransferase[All Fields])) OR (HYAL1[All Fields] OR (("hyaluronoglucosaminidase"[MeSH Terms] OR "hyaluronoglucosaminidase"[All Fields] OR "hyaluronidase"[All Fields]) AND 1[All Fields])) OR (IDS[All Fields] OR ("iduronate sulfatase"[MeSH Terms] OR ("iduronate"[All Fields] AND "sulfatase"[All Fields]) OR "iduronate sulfatase"[All Fields] OR "iduronate 2 sulfatase"[All Fields])) OR (IDUA[All Fields] OR (("iduronidase"[MeSH Terms] OR "iduronidase"[All Fields]) AND alpha-L[All Fields])) OR (KCTD7[All Fields] OR (("potassium channels"[MeSH Terms] OR ("potassium"[All Fields] AND "channels"[All Fields]) OR "potassium channels"[All Fields] OR ("potassium"[All Fields] AND "channel"[All Fields]) OR "potassium channel"[All Fields]) AND tetramerization[All Fields] AND ("protein domains"[MeSH Terms] OR ("protein"[All Fields] AND "domains"[All Fields]) OR "protein domains"[All Fields] OR "domain"[All Fields]) AND containing[All Fields] AND 7[All Fields])) OR (LAMP2[All Fields] OR ("lysosomal-associated membrane protein 2"[MeSH Terms] OR "lysosomal-associated membrane protein 2"[All Fields] OR "lysosomal associated membrane protein 2"[All Fields])) OR (LIPA[All Fields] OR (("lipase"[MeSH Terms] OR "lipase"[All Fields]) AND lysosomal[All Fields] AND ("acids"[MeSH Terms] OR "acids"[All Fields] OR "acid"[All Fields]) AND type[All Fields])) OR MAN2B1[All Fields] OR (MANBA[All Fields] OR (("mannosidases"[MeSH Terms] OR "mannosidases"[All Fields] OR "mannosidase"[All Fields]) AND ("beta"[All Fields]))) OR (MCOLN1[All Fields] OR (mucolipin[All Fields] AND 1[All Fields])) OR MFSD8[All Fields] OR (NAGA[All Fields] OR ("alpha-n-acetylgalactosaminidase"[MeSH Terms] OR "alpha-n-acetylgalactosaminidase"[All Fields] OR "alpha n acetylgalactosaminidase"[All Fields])) OR (NAGLU[All Fields] OR (N-acetyl-alpha-glucosaminidase[All Fields])) OR (NEU1[All Fields] OR (("neuraminidase"[MeSH Terms] OR "neuraminidase"[All Fields]) AND 1[All Fields])) OR (NPC1[All Fields] OR (("npc"[All Fields]) AND ("cytoplasm"[MeSH Terms] OR "cytoplasm"[All Fields] OR "intracellular"[All Fields]) AND ("cholesterol"[MeSH Terms] OR "cholesterol"[All Fields]) AND ("membrane transport proteins"[MeSH Terms] OR ("membrane"[All Fields] AND "transport"[All Fields] AND "proteins"[All Fields]) OR "membrane transport proteins"[All Fields] OR "transporter"[All Fields]) AND 1[All Fields])) OR (NPC2[All Fields] OR (("npc"[All Fields]) AND ("cytoplasm"[MeSH Terms] OR "cytoplasm"[All Fields] OR "intracellular"[All Fields]) AND ("cholesterol"[MeSH Terms] OR "cholesterol"[All Fields]) AND ("membrane transport proteins"[MeSH Terms] OR ("membrane"[All Fields] AND "transport"[All Fields] AND "proteins"[All Fields]) OR "membrane transport proteins"[All Fields] OR "transporter"[All Fields]) AND 2[All Fields])) OR (PPT1[All Fields] OR ("palmitoyl-protein thioesterase"[Supplementary Concept] OR "palmitoyl-protein thioesterase"[All Fields] OR "palmitoyl protein thioesterase 1"[All Fields])) OR (PSAP[All Fields] OR prosaposin[All Fields]) OR SCARB2[All Fields] OR (SGSH[All Fields] OR ("N-sulfoglucosamine sulfohydrolase"[Supplementary Concept] OR "N-sulfoglucosamine sulfohydrolase"[All Fields] OR "n sulfoglucosamine sulfohydrolase"[All Fields])) OR (SLC17A5[All Fields] OR (solute[All Fields] AND ("carrier state"[MeSH Terms] OR ("carrier"[All Fields] AND

"state"[All Fields]) OR "carrier state"[All Fields] OR "carrier"[All Fields]) AND ("family"[MeSH Terms] OR "family"[All Fields]) AND 17[All Fields] AND member[All Fields] AND 5[All Fields])) OR (SMPD1[All Fields] OR (("sphingomyelin phosphodiesterase"[MeSH Terms] OR ("sphingomyelin"[All Fields] AND "phosphodiesterase"[All Fields]) OR "sphingomyelin phosphodiesterase"[All Fields]) AND 1[All Fields])) OR ST3GAL5[All Fields] OR (SMUF1[All Fields] OR (("sulfatases"[MeSH Terms] OR "sulfatases"[All Fields] OR "sulfatase"[All Fields]) AND modifying[All Fields] AND factor[All Fields] AND 1[All Fields])) OR (TPP1[All Fields] OR ("tripeptidyl-peptidase 1"[Supplementary Concept] OR "tripeptidyl-peptidase 1"[All Fields] OR "tripeptidyl peptidase 1"[All Fields]))

)

AND

("biomarkers"[MeSH Terms] OR "biomarkers"[All Fields] OR "biomarker"[All Fields] OR ("biological"[All Fields] AND "marker"[All Fields]) OR ("biological"[All Fields] AND "markers"[All Fields]) OR "biological marker"[All Fields] OR "biological markers"[All Fields] OR "blood"[Subheading] OR "blood"[All Fields] OR "blood"[MeSH Terms] OR "serum"[MeSH Terms] OR "serum"[All Fields] OR "plasma"[MeSH Terms] OR "plasma"[All Fields] OR "cerebrospinal fluid"[Subheading] OR ("cerebrospinal"[All Fields] AND "fluid"[All Fields]) OR "cerebrospinal fluid"[All Fields] OR "cerebrospinal fluid"[MeSH Terms] OR ("cerebrospinal"[All Fields] AND "fluid"[All Fields]) OR "urine"[Subheading] OR "urine"[All Fields] OR "urine"[MeSH Terms] OR "saliva"[MeSH Terms] OR "saliva"[All Fields] OR "skin"[MeSH Terms] OR "skin"[All Fields] OR "exosomes"[MeSH Terms] OR "exosomes"[All Fields] OR "exosome"[All Fields] OR "imaging"[All Fields])

AND

("parkinson disease"[MeSH Terms] OR ("parkinson"[All Fields] AND "disease"[All Fields]) OR "parkinson disease"[All Fields] OR "parkinson's"[All Fields])
